# Epigenetic control of the basal-like gene expression profile via Interleukin-6 in breast cancer cells

**DOI:** 10.1186/1476-4598-9-300

**Published:** 2010-11-23

**Authors:** Laura D'Anello, Pasquale Sansone, Gianluca Storci, Valentina Mitrugno, Gabriele D'Uva, Pasquale Chieco, Massimiliano Bonafé

**Affiliations:** 1 Center for Applied Biomedical Research (CRBA), St. Orsola-Malpighi University Hospital, via Massarenti 9, 40138 Bologna, Italy; 2 Department of Experimental Pathology, University of Bologna, via San Giacomo 14, 40126 Bologna, Italy; 3 Department of Pharmacology and Toxicology, University of Bologna, Via Irnerio 48- s40126 Bologna, Italy

## Abstract

**Background:**

Basal-like carcinoma are aggressive breast cancers that frequently carry p53 inactivating mutations, lack estrogen receptor-α (ERα) and express the cancer stem cell markers CD133 and CD44. These tumors also over-express Interleukin 6 (IL-6), a pro-inflammatory cytokine that stimulates the growth of breast cancer stem/progenitor cells.

**Results:**

Here we show that p53 deficiency in breast cancer cells induces a loss of methylation at *IL-6 *proximal promoter region, which is maintained by an IL-6 autocrine loop. IL-6 also elicits the loss of methylation at the *CD133 *promoter region 1 and of *CD44 *proximal promoter, enhancing *CD133 *and *CD44 *gene transcription. In parallel, IL-6 induces the methylation of estrogen receptor (ERα) promoter and the loss of ERα mRNA expression. Finally, IL-6 induces the methylation of *IL-6 *distal promoter and of *CD133 *promoter region 2, which harbour putative repressor regions.

**Conclusion:**

We conclude that IL-6, whose methylation-dependent autocrine loop is triggered by the inactivation of p53, induces an epigenetic reprogramming that drives breast carcinoma cells towards a basal-like/stem cell-like gene expression profile.

## Background

Basal-like tumors are aggressive estrogen receptor-α (ERα) negative breast carcinomas that have been identified due to their peculiar gene expression profile [1-3]. Such tumors display a stem cell-like gene expression profile, including the over-expression of cancer stem cells (CSCs) markers, such as CD133 [1,4] and CD44 [5-9]. CD44 and CD133 are also over-expressed in multicellular spheroids (called mammospheres), derived from breast cancer tissues and cell lines [10,11]. Mammosphere-forming subpopulation of breast cancer cells are endowed with highly enhanced tumor-initiating capability and with resistance to cancer therapy, and are currently dubbed as breast CSCs [12-14]. Similarly to basal-like tumors, breast CSCs lack ERα expression [1-3,15,16]. Basal-like tumors also over-express the pro-inflammatory cytokine Interleukin-6 (IL-6), a potent growth factor for breast cancer cells that enhances mammospheres growth capacity and malignant features in a paracrine/autocrine fashion [3,5,10].

Basal-like breast cancers carry inactivating mutations of the tumor suppressor *p53 *in about 80% of cases [1-3]. It has been reported that p53 represses the expression of IL-6 and CD44, via direct promoter binding [17,18]. p53 exerts various check-point activities, including the repression of gene transcription through the methylation of DNA promoters, a mechanism of epigenetic regulation catalyzed by DNA (cytosine-5)-methyltransferases at CpG dyads dinucleotides [19-21]. Interestingly, basal-like cells and tissues exhibit a peculiar promoter methylation pattern and over-express genes involved in genomic DNA and histone methylation [22-25].

We therefore hypothesized that *IL-6*, *CD44*, *CD133 *and *ERα *take part to the basal-like gene expression profile throughout the epigenetic modification of their promoter regions.

We show that p53 deficiency induces the loss of methylation at the *IL-6 *promoter. This phenomenon starts an autocrine IL-6 loop that favours the loss of methylation at *IL-6, CD44 *and *CD133 *promoter 1, as well as the gain of methylation at ERα promoter. In parallel, the expression of *IL-6*, *CD44 *and *CD133 *is enhanced, and that of *ERα *is blunted. Moreover, IL-6 induces the methylation of IL-6 distal promoter and of CD133 promoter region 2, which contain putative repressor binding sites.

We conclude that p53 deficiency induces an IL-6 dependent epigenetic reprogramming that drives breast carcinoma cells towards a basal-like/stem cell-like gene expression profile.

## Materials and methods

### Chemicals and reagents

αIL-6, a monoclonal antibody that blocks the IL-6 receptor/ligand interaction [10], recombinant human IL-6, 4-hydroxytamoxifen (4OHT, Tamoxifen) and the demethylathing agent 5-aza-2'-deoxycytidine (5azadC) were purchased from Sigma (Sigma, St-Louis, MO, USA).

### Cell cultures

MCF-7 cells (carrying wild type p53) were cultured in RPMI medium supplemented with fetal bovine serum (FBS 10%), 100 IU/mL penicillin, and 100 μg/mL streptomycin. MCF-7 cells stably transduced with pBabe retroviral vector encoding p53 dominant-negative mini-protein were cultured as previously described [26,27]. MCF-7 derived mammospheres were obtained as previously described [4,10,27]. P53 deficient MDA-MB231 breast cancer cell line (carrying R280K mutation) [5] were cultured in Dulbecco's modified Eagle's medium (DMEM) supplemented with fetal bovine serum (FBS 10%), 100 IU/mL penicillin, and 100 μg/mL streptomycin.

### RNA extraction and RT-PCR analysis

Total RNA was extracted from cultured cells using the RNA-extracting reagent TRIzol (Invitrogen) according to the manufacturer's instructions.

Reverse transcription reaction was performed in a 20 μl volume with 2 μg of total RNA using the M-MLV Reverse Transcriptase, following the manufacturer's protocol. Oligo-(dT) 12-18 primers (Invitrogen) were used for the first strand synthesis. PCR primers (Additional file 1 Table 1) and reagents were purchased from Invitrogen.

### Transient RNA interference

Double-strand RNA oligonucleotides (siRNA) directed against p53, IL-6 and ERα mRNA (Stealth validated RNAi DuoPaks), and appropriate control scrambled siRNA, were purchased from Invitrogen. siRNAs were transfected to adherent MCF-7 cells (10^5 ^cells in a 3-cm^2^well) at a concentration of 1 μg/well using Lipofectamine 2000 (Invitrogen).

### DNA methylation assay

Methylation specific PCR was performed as previously described [28]. DNA was extracted with phenol/chloroform (Sigma) and Proteinase K (Invitrogen, Carlsbad, CA, USA) and was bisulphite-modified with EZ-Methylation Gold-Kit (Zymo Research Corporation Orange, CA U.S.A) according to the manufacturer's instructions. Bisulphite modified DNA was amplified with primers designed using design Methyl Express^® ^Software v1.0 (Applied Biosystems Foster City, CA USA) and Beacon Designer 3.0 (Premier Biosoft International, Palo Alto CA USA; Additional file 2 Table 2). PCR primers and reagents were purchased from Invitrogen. PCR protocols were performed as follows: pre-denaturation step at 95°C for 2 min, 35 to 40 cycles of denaturation at 95°C for 30 sec, annealing at the appropriate temperature for 30 sec, extension at 72°C for 1 min; final extension at 72°C for 7 min. Sequence of genomic DNA promoters are: *IL-6 *[GenBank: M18403], *CD133 *p1 and p2 promoters [GenBank: ay275524], *CD44 *[GenBank: M59040], *ERα *[GenBank: X03635] (Additional file 3 Figure S1). Search for transcriptional factor binding sites was performed by TESS: Transcription Element Search Software on the WWW, Jonathan Schug and G. Christian Overton, Technical Report CBIL-TR-1997-1001-v0.0 Computational Biology and Informatics Laboratory, School of Medicine University of Pennsylvania, 1997 URL: http://www.cbil.upenn.edu/tess.

### Fluorometry

Amplified fragments were resolved onto a 1.8% agarose gel with ethidium bromide.

Gels were imaged with FluorSMultiImager (Bio-Rad, Hercules, CA) using UV excitation and a barrier filter of 520 nm. Emission of amplified bands was analysed with QuantityOne 4.6.6 software (Bio-Rad) using the same reading frame. Given values are ratios between the Unmethylated "U" and Methylated "M" band emissions of the same fragment. Experimental values were normalized with the U/M ratio of control fragments to which a value of 1 was assigned.

### Luciferase Assay

DNA transfection of MCF-7 cells was performed with Lipofectamine 2000 (Invitrogen). One day before transfection, the cells were seeded at a density of 1.5 × 10^5 ^cells/well on 6-well plates and transfected with 1 μg of luciferase reporters driven by either p53 responsive elements (Stratagene, La Jolla, CA USA), or the -2,161 to -41bp IL6 promoter fragment (kindly provided by W. L. Farrar, NCI-Frederick Cancer Research and Development Center, USA) [29], or the -1192bp to +10b CD133 promoter 1 fragment (Kindly provided by K. Tabu, Department of Stem Cell Regulation, Medical Research Institute, Tokyo Medical and Dental University, Japan) [30]. IL-6 promoter reporter activity was also tested when co-transfected with 1 μg of IRF-1 or IRF-2 encoding pCAG vectors (kindly provided by T. Taniguchi, Department of Immunology, Graduate School of Medicine and Faculty of Medicine, University of Tokyo, Japan).

[31]. Firefly Luciferase was normalized by co-trasfecting 10 ng of Thymidine Kinase Renilla Luciferase reporter (Promega Corporation, Madison Wisconsin USA). All luciferase assays were performed in triplicates following manufacturer's instructions (Promega).

### Luciferase assay on *in vitro *methylated Luciferase reporter

*In vitro *plasmid DNA methylation was performed as previously described [30]. Briefly, 4 μg of -1192bp to +10b CD133 promoter 1 fragment [30] were incubated with 5 units of SssI (CpG) methylase (Zymo Research Corporation) for 4 h, per 1 μg of plasmid DNA in presence (methylated) or absence (unmethylated) of 0.64 mM S-adenosylmethionine. After phenol purification, equal amounts of methylated and unmethylated reporter constructs were assessed by digestion with the methylation-sensitive restriction enzyme HpaII (Promega) and were then assessed in a luciferase assay as above described.

### Western blot

Protein concentration was determined by Protein Assay reagent (Bio-Rad, Richmond, CA, USA). Sixty μg of proteins were separated by SDS-PAGE and transferred to a nitrocellulose filter that was subsequently incubated with TBS buffer containing 5% dried nonfat-milk for 2 hour at room temperature (RT). Filters were probed with mouse monoclonal antibodies to p53 (DO-1 Santa Cruz Biotechnology, Santa Cruz, CA, USA), human STAT-3 and phosphorylated STAT-3 (Cell Signaling Technology, Danvers, MA USA) and to β-Actin (Santa Cruz). Bound antibodies were detected with peroxidase-labelled goat antibody to mouse or rabbit IgG and visualized by enhanced chemiluminescence reagents (Amersham Pharmacia Biotech, Freiburg, Germany).

### IL-6 ELISA Assay

Quantitative detection of human IL-6 in cell culture supernatants was determined by Human IL-6 ELISA kit (Immunological sciences, Roma, Italy). The assay was performed in duplicates, following manufacturer's instructions. The plate was read by Thermo Labsystems Multiskan Ascent Photometric plate reader for 96 and 384 well plates (American Instrument Exchange, Inc., Haverhill, MA, USA).

### Immunofluorescence

Adherent cells (seeded at a density of 5 × 10^4^) and mammospheres were fixed with 4% paraformaldehyde for 10 minutes, permeabilized with Triton X-100 0.2% for 30 minutes and incubated with anti-CD44 mouse monoclonal antibody (1:250, Cell Signaling) in PBS-1%BSA for 1 h at 37°C. After PBS washing, cells and mammospheres were incubated with anti-mouse fluorescein-conjugated antibody (1:250, Santa Cruz) in PBS-1% BSA for 45 min at 37°C in dark room and with DAPI solution (1:1000, 4',6-diamidino-2-phenylindole, KPL, Gaithersburg, MD USA) for 15 min, and mounted in anti-fade Pro long reagent mounting medium (Molecular Probes Inc, Eugene, Oregon, USA). Images were captured using a Leica DMI 6000B inverted microscope (Leica Microsystems GmbH, Wetzlar, Germany).

### Statistical analysis

Data were analyzed by t-Student test (SPSS, Chicago, IL, USA). Data were considered significant when p < 0.05.

## Results

### Reduced *IL-6 *proximal promoter methylation and high IL-6 expression in breast cancer cells carrying inactivated p53

**Figure 1:**
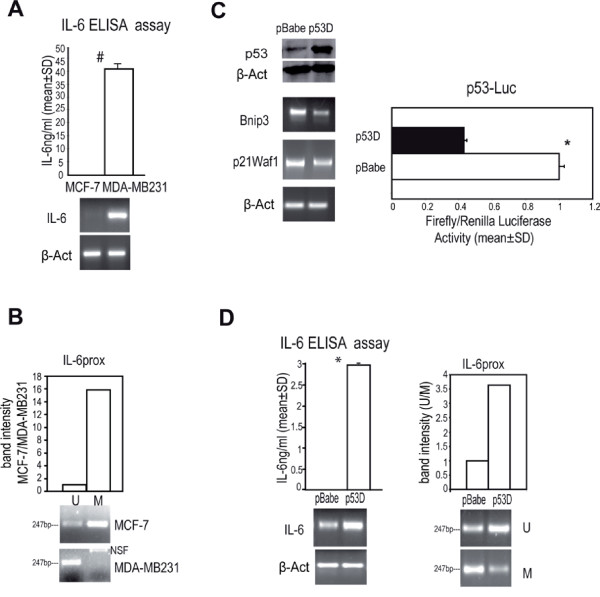
**p53 inactivation up-regulates IL-6 expression and induces the loss of methylation at *IL-6 *proximal promoter**. a) IL-6 ELISA assay and RT-PCR analysis of IL-6 mRNA level in MCF-7 and MDA-MB231 cells; b) quantitative evaluation of *IL-6prox *methylation-specific PCR analysis (MS-PCR) in MCF-7 and MDA-MB231 cells. "U" stands for Unmethylated and "M" Methylated specific PCR product; c) Western blot analysis of p53 protein level, RT-PCR analysis of Bnip3 and p21Waf1 mRNA level and luciferase assay of p53 responsive reporter plasmid (p53-Luc) in MCF-7 cells transduced with empty (pBabe) or p53 mini-protein (p53D) retroviral vector; d) IL-6 ELISA assay, RT-PCR analysis of IL-6 mRNA level and quantitative evaluation of *IL-6prox *MS-PCR analysis in pBabe/p53 D cells. β-Actin was assessed as quantitative control for RT-PCR and Western Blot. Student t test, *p < 0.05, ^#^p < 0.005. NSF: Non Specific Fragment

In keeping with previous observations [5,31], significantly higher IL-6 mRNA and secreted IL-6 protein levels were found in MDA-MB231 cells (that carry an R280K inactivating mutation in p53 gene) [5] compared to p53 wild type MCF-7 cells (Figure 1A). Such a high IL-6 expression in MDA-MB231 cells was correlated with the lack of methylation at the -55;+189bp IL-6 promoter region (referred to as *IL-6prox*, Additional file 3 Figure S1 A). To assess the role of p53 inactivation on IL-6 expression and *IL-6prox *methylation, we transduced MCF-7 cells with a retroviral vector encoding a truncated dominant negative p53 mini-protein [27]. Such cells (named as p53D) disclosed an accumulation of p53 protein, coupled with reduced p53 activity that led to a decrease in the mRNA level of p53 responsive genes (Bnip3, p21Waf1) and to a reduction of p53 dependent luciferase reporter activity (Figure 1C). According to our hypothesis, p53 D cells showed high IL-6 mRNA and protein secretion and exhibited a decrease in *IL-6prox *methylation compared to empty vector transfected cells (Figure 1D). As a further proof of the role of p53 in the modulation of IL-6 expression and *IL-6prox *methylation level, we transfected MCF-7 cells with p53 specific siRNA (sip53) and we found an increase in IL-6 mRNA level, as well as a reduction of methylation at *IL-6prox *(Additional file 4 Figure S2). These data suggest that p53 inactivation enhances IL-6 expression via an epigenetic modification of the proximal promoter region in breast cancer cells.

### Decreased methylation at *IL-6prox *promoter region is induced and maintained by exogenous or autocrine IL-6

**Figure 2:**
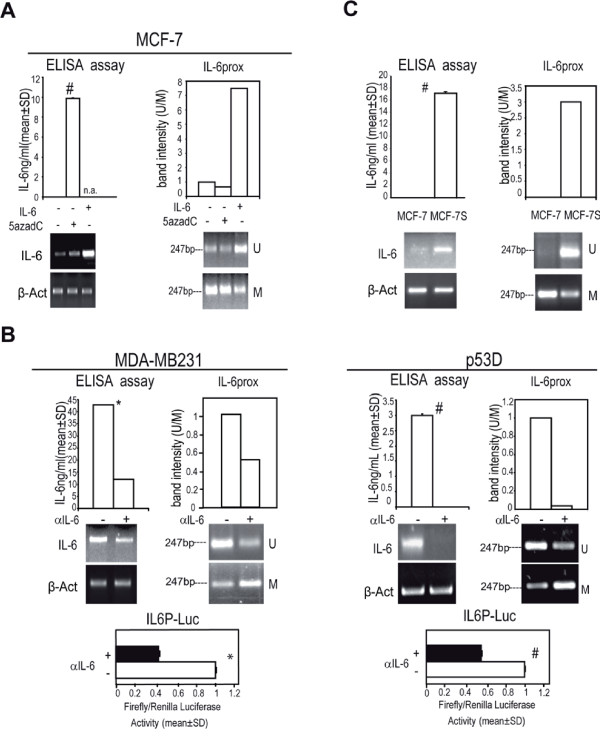
**Reduced methylation at *IL-6prox *and induction of IL-6 expression by exogenous or autocrine IL-6**. a) IL-6 ELISA assay, RT-PCR analysis of IL-6 mRNA level and quantitative evaluation of *IL-6prox *MS-PCR analysis in MCF-7 cells in presence/absence of IL-6 (10 ng/ml, 48 h) or 5AzadCytidine (5azadC, 15 μM, 48 h); b) IL-6 ELISA assay, RT-PCR analysis of IL-6 mRNA level, quantitative evaluation of *IL-6prox *MS-PCR analysis and IL-6 promoter driven luciferase assay (IL6P-Luc) in MDA-MB231cells and p53 D cells in presence/absence to αIL-6 (1.5 μg/ml, 48 h); C) IL-6 ELISA assay, RT-PCR analysis of IL-6 mRNA level and quantitative evaluation of *IL-6prox *MS-PCR analysis in MCF-7 derived mammospheres (MCF-7S) or MCF-7 adherent cells; β-Actin was assessed as quantitative control for RT-PCR analysis. Student t test, *p < 0.05; ^#^p < 0.005. n.a.: not assessed.

We have previously observed that IL-6 exposure in breast cancer cells elicits the up-regulation of its own mRNA [10]. We here confirmed that both the cytokine itself and 5-AzaCytidine (5azadC) administration elicited IL-6 expression and a parallel decrease of *IL-6prox *methylation (Figure 2A). Accordingly, exposure of MDA-MB231 and p53 D cells to αIL-6 (an antibody which blocks IL-6/IL-6 receptor interaction and activity, Additional file 5 Figure S3) led to a down-regulation of IL-6 expression, to an increase in methylation at *IL-6prox *and to a decrease in IL6 promoter driven-luciferase activity (IL6P-Luc, Figure 2B). As a support for the role of IL-6 in the modulation of *IL-6prox *methylation status, we observed that MCF-7 derived mammospheres (MCF-7S), which express high levels of IL-6 mRNA and secreted protein show a reduced methylation at *IL-6prox *compared to MCF-7 adherent cells (Figure 2C). Hence, IL-6 elicits its own mRNA expression and proximal promoter loss of methylation in breast cancer cells.

### Up-regulation of CD133, CD44 and down-regulation of ERα expression mRNA by IL-6 and 5azadC in breast cancer cells

**Figure 3:**
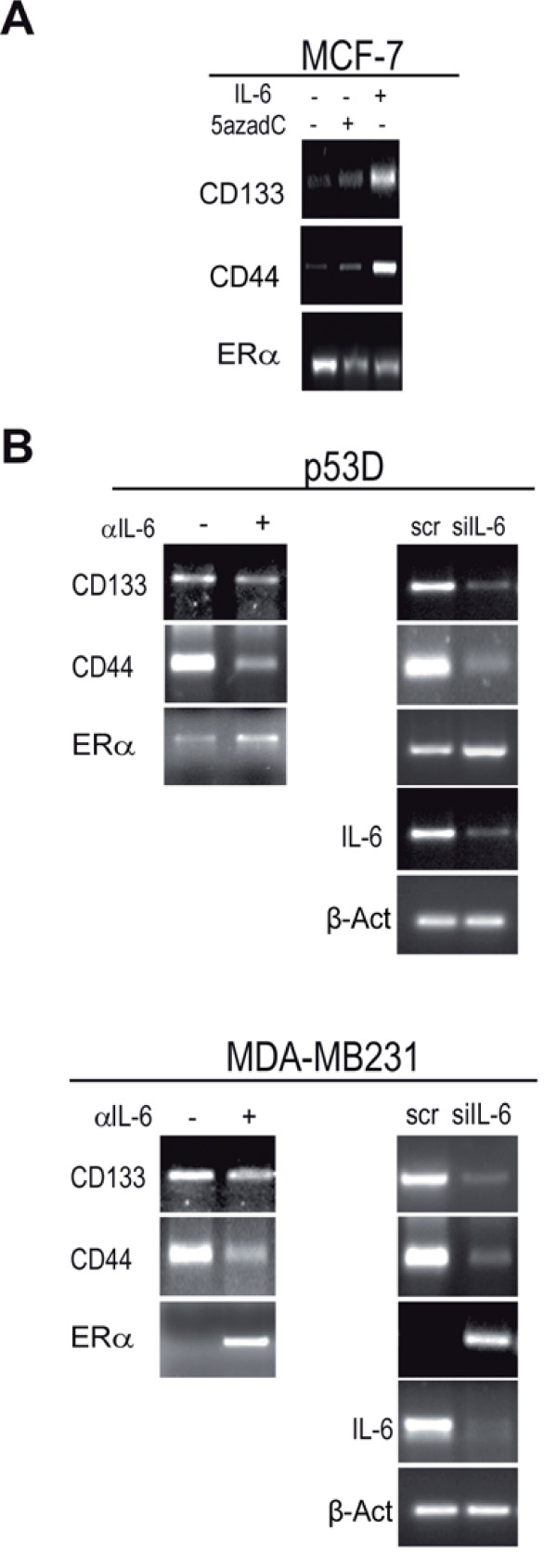
**Regulation of CD133, CD44 and ERα mRNA by exogenous or autocrine IL-6**. RT-PCR analysis of CD133, CD44, ERα mRNA level in: a) MCF-7 cells in presence/absence of IL-6 (10 ng/ml, 48 h) or 5azadC (15 μM, 48 h), b) p53 D cells and MDA-MB231 cells in presence/absence of αIL-6 (1.5 μg/ml, 48 h) or transfected with scr/siIL-6 (1 μg, 48 h). β-Actin of panel a) and b) are reported in panels 2a and 2b, respectively.

We then aimed at investigating whether other genes involved in the basal-like gene profile are regulated by IL-6. The administration of IL-6 or 5azadC to MCF-7 cells up-regulated CD133 and CD44 and down-regulated ERα mRNA levels (Figure 3A).

Moreover, the administration of αIL-6 or an IL-6 specific siRNA (siIL-6) to p53 D and MDA-MB231 cells down-regulated CD133 and CD44 mRNA and up-regulated ERα mRNA expression (Figure 3B). These data prompted us to test the hypothesis that CD133, CD44 and ERα may be epigenetically controlled by IL-6.

### Decrease in methylation at *CD133 *proximal promoter by exogenous or autocrine IL-6

**Figure 4:**
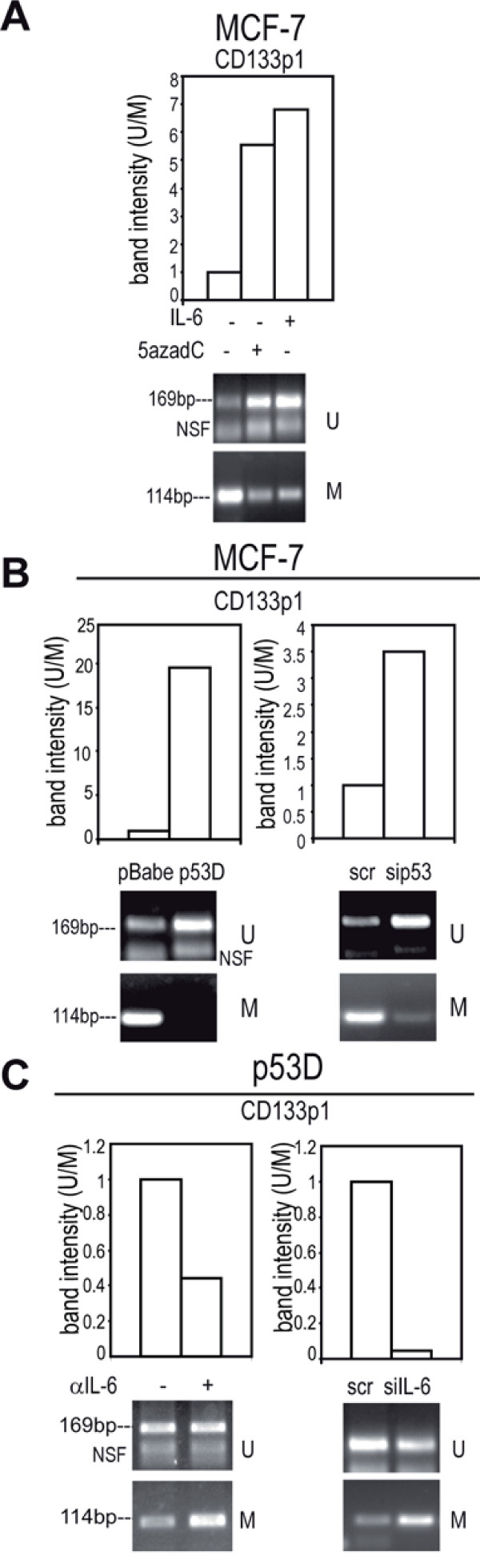
**Decrease in methylation at *CD133 *promoter 1 (CD133p1) by exogenous or autocrine IL-6**. Quantitative evaluation of *CD133*p1 MS-PCR analysis in: a) MCF-7 cells in presence/absence of IL-6 (10 ng/ml, 48 h) or 5azadC (15 μM, 48 h); b) pBabe/p53 D and scr/sip53 transfected (1 μg, 48 h) MCF-7 cells, c) p53 D cells in presence/absence of αIL-6 (1.5 μg/ml, 48 h) or transfected with scr/siIL-6 (1 μg, 48 h). NSF Non Specific Fragment.

We observed that the administration of IL-6 or 5azadC reduced the methylation at CD133 *promoter 1 *(*CD133*p1, Additional file 3 Figure S1 B and Figure 4A). Accordingly, IL-6 triggered the activity of *CD133*p1 driven luciferase reporter, which was inhibited by *in vitro *methylation (Additional file 6 Figure S4 A). A reduction in methylation at *CD133*p1 was then observed in IL-6 expressing MCF-7 S compared to adherent MCF-7 cells (Additional file 6 Figure S4 B). Moreover, a substantial decrease of *CD133*p1 methylation was found in p53 D, sip53 transfected (Figure 4B) and MDA-MB231 cells (Additional file 6 Figure S4 C). The administration of αIL-6 or the transfection of siIL-6 induced a gain of methylation of *CD133*p1 in p53 D (Figure 4C) and MDA-MB231 cells (Additional file 6 Figure S4 D). These data suggest that IL-6 elicits an epigenetic control on *CD133*p1 promoter.

### Reduced methylation at *CD44 *proximal promoter by exogenous or autocrine IL-6

**Figure 5:**
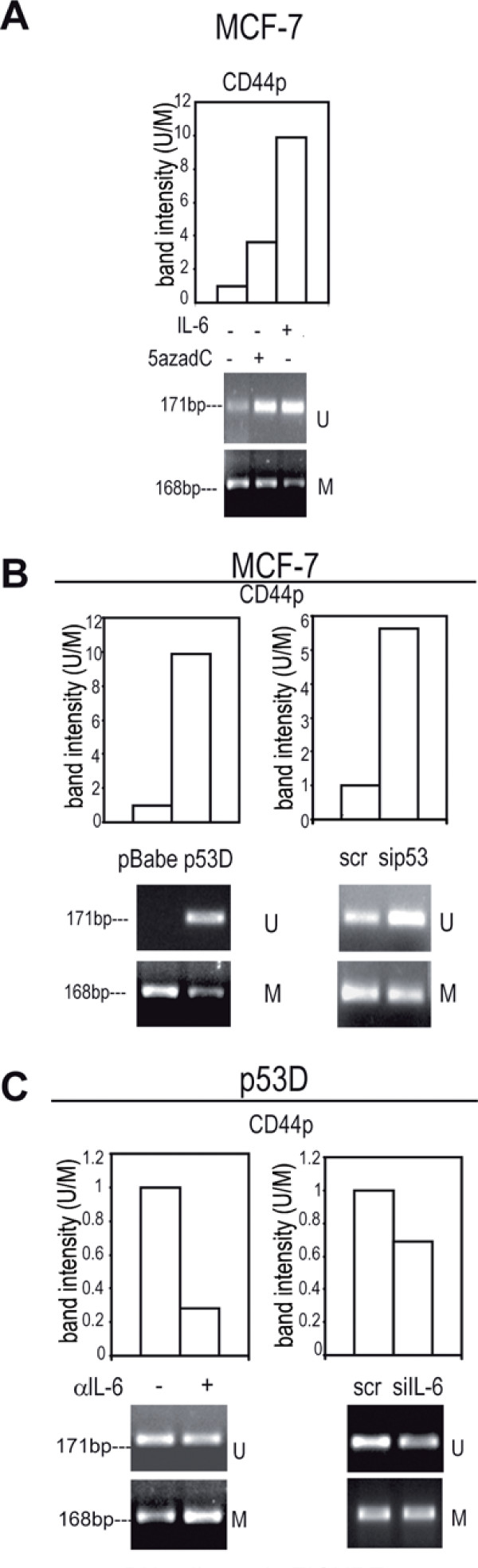
**Reduction of methylation at *CD44 proximal *promoter (CD44p) by exogenous or autocrine IL-6**. Quantitative evaluation of *CD44p *MS-PCR analysis in: a) MCF-7 cells in presence/absence of IL-6 (10 ng/ml, 48 h) or 5azadC (15 μM, 48 h); b) pBabe/p53 D and scr/sip53 transfected (1 μg, 48 h) cells, c) p53 D cells in presence/absence of αIL-6 (1.5 μg/ml, 48 h) or transfected with scr/siIL-6 (1 μg, 48 h).

We observed that the administration of IL-6 or 5azadC to MCF-7 cells decreased methylation at CD44 proximal promoter (*CD44*p, Additional file 3 Figure S1 C, Figure 5A). Reduced *CD44*p methylation was also observed in MCF-7 S compared to adherent MCF-7 cells (Additional file 7 Figure S5 A). Moreover, we found a reduction of *CD44*p methylation in p53 D, sip53 transfected (Figure 5B) and MDA-MB231cells (Additional file 7 Figure S5 B), a phenomenon that was reversed by αIL-6 and siIL-6 administration (Figure 5C and Additional file 7 Figure S5 C). These data suggest that IL-6 elicits an epigenetic regulation of *CD44 *gene.

### Increased methylation at ER*α *promoter by exogenous or autocrine IL-6

**Figure 6:**
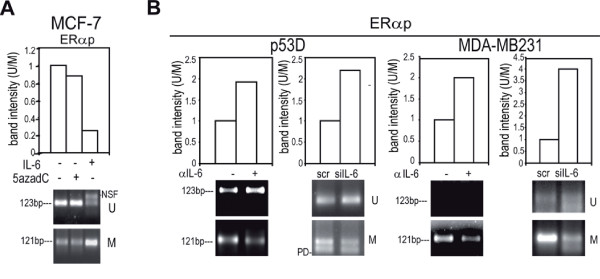
**Gain of *ERα *promoter (*ERαp*) methylation by autocrine or exogenous IL-6**. Quantitative evaluation of *ERαp *MS-PCR analysis in: a) MCF-7 cells in presence/absence of IL-6 (10 ng/ml, 48 h) or 5azadC (15 μM, 48 h); b) p53 D and MDA-MB231 cells in presence/absence of αIL-6 (1.5 μg/ml, 48 h) or scr/siIL-6 (1 μg, 48 h). PD: Primer Dimers, NSF Non Specific Fragment.

We also found that IL-6 or 5azadC elicited a gain of methylation at *ERα *promoter (*ERαp*, Additional file 3 Figure S1 D, Figure 6A). Accordingly, MDA-MB231 and p53 D exposed to αIL-6 or transfected with siIL-6 showed a decrease of *ERαp *methylation (Figure 6B). These data suggest that an IL-6 dependent epigenetic mechanism may take part to ER*α *down-regulation in basal-like carcinoma.

### Increased methylation at IL-6 distal promoter and at CD133 promoter region 2 by exogenous or autocrine IL-6

**Figure 7:**
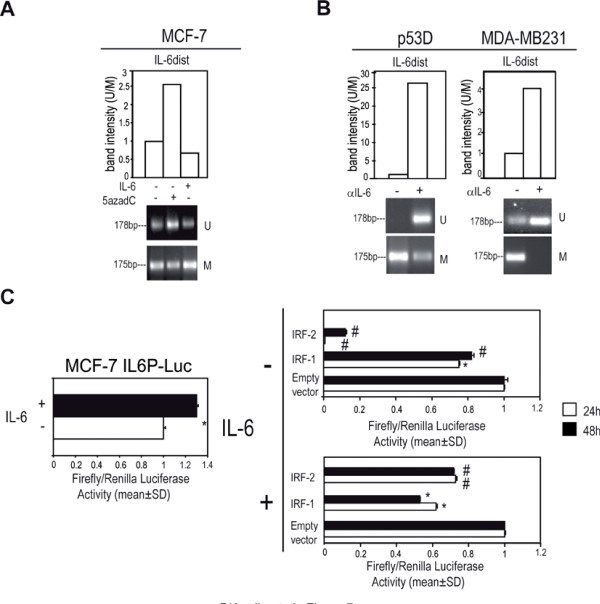
***IL-6dist *gains methylation in response to IL-6 and contains putative repressive IRF-1/IRF-2 binding sites**. Quantitative evaluation of *IL-6dist *MS-PCR analysis in: a) MCF-7 cells in presence/absence of IL-6 (10 ng/ml, 48 h) or 5azadC (15 μM, 48 h), b) p53 D and MDA-MB231cells in presence/absence of αIL-6 (1.5 μg/ml, 48 h); c) Luciferase assay of MCF-7 cells transfected with IL6P-Luc in presence/absence of IL-6 (10 ng/ml), and pIRF-1 or pIRF-2 (1 μg each, 24/48 h) in presence/absence of IL-6 (10 ng/ml, lower panel). Student t test, *p < 0.05; ^#^p < 0.005.

In basal-like cancer, complex changes in genomic DNA methylation pattern have been reported [22-25]. In this regard, we found that the administration of IL-6 to MCF-7 cells elicited an increase of methylation at *IL-6 *distal promoter (*IL-6dist*, Additional file 3 Figure S1 E and Figure 7A). Moreover, αIL-6 administration to p53 D and MDA-MB231 cells reduced methylation at *IL-6dist *(Figure 7B). Such region harbours putative consensus binding sites for Interferon regulatory transcription factor 1/2 (IRF-1/2, Additional file 3 Figure S1 E) that can act as repressor for gene transcription [32]. In this regard, we observed that IL6P-Luc activity was reduced when co-transfected with vectors encoding IRF-1/2 proteins (Figure 7C).

**Figure 8:**
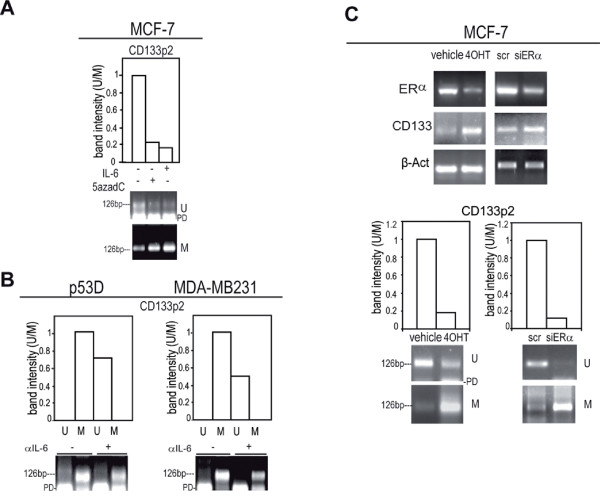
***CD133p2 *gains methylation in response to IL-6 and contains putative repressive *ERα *binding sites**. Quantitative evaluation of *CD133*p2 MS-PCR analysis in: a) MCF-7 cells in presence/absence of IL-6 or 5azadC (10 ng/ml and 15 μM, 48 h, respectively), b) p53 D and MDA-MB231 cells in presence/absence of αIL-6 (1.5 μg/ml, 48 h); c) RT-PCR analysis of ERα and CD133 mRNA level and quantitative evaluation of *CD133p2 *MS-PCR analysis in MCF-7 cells exposed to Tamoxifen (4OHT, 3 μM, 48 h or vehicle) or to scr or ERα specific siRNA (siERα, 1 μg, 48 h); β-Actin was assessed as quantitative control in RT-PCR analysis. PD: primer Dimers.

We also observed that methylation at *CD133 promoter 2 *region (Additional file 3 Figure S1 F) was increased following IL-6 exposure in MCF-7 cells (Figure 8A). Accordingly, *CD133p2 *methylation was decreased in p53 D and MDA-MB231 cells following the administration of αIL-6 (Figure 8B). Due to the presence of several putative ERα binding sites at *CD133p2 *(Additional file 3 Figure S1 F), we hypothesized that ERα exerts a repressive activity on CD133 expression. In line with this hypothesis, when ERα positive MCF-7 cells were exposed to the ERα inhibitory drug Tamoxifen or to an ERα specific siRNA an up-regulation of CD133 mRNA, paralleled by a substantial gain of methylation at *CD133p2 *were observed (Figure 8C).

These data indicate that IL-6 induces complex changes in genomic DNA, including the methylation of putative repressor regions.

## Discussion

In this investigation, we show that the abrogation of p53 function, a distinctive feature of basal-like breast carcinomas, is functionally associated with the loss of methylation at the *IL-6 *proximal promoter, a crucial region for *IL-6 *gene expression [29,31]. In p53 deficient breast cancer cells, the loss of methylation at *IL-6 *proximal promoter is maintained by an autocrine loop which further takes to an *IL-6 *dependent loss of methylation at *CD133 *and *CD44 *promoters. These epigenetic modifications also occur when recombinant IL-6 is exogenously administered to the cells. In addition, IL-6 administration elicits the gain of methylation at *ERα *promoter, whose epigenetic regulation is of primary importance in breast cancer biology [1-3,33,34]. Paralleling these phenomena, we observed an up-regulation of CD44 and CD133 mRNA coupled with a down-regulation of ER*α *mRNA. Current literature indicates that the above pattern of gene expression is proper of basal-like tumors and CSCs [2,3,15,16,27].

Previously, IL-6 itself was found to be over-expressed in basal-like tumors and CSCs, and to enhance mammosphere forming capacity [10,35]. Accordingly, we report in the present work that MCF-7 derived mammospheres show an over-expression of *IL-6*, *CD133 *and *CD44 *genes, as a possible consequence of the loss of promoter methylation. Considering the close relationship existing between mammospheres and CSCs [4,10-14], these findings support the very recent observation [35] that IL-6 driven epigenetic changes are associated with CSCs features in breast cancer cells. In line with this reasoning, both CD133 and CD44 have been previously reported to be regulated by promoter methylation [30,31,36]. Moreover, multiple epigenetic promoter modifications have been found to control IL-6 gene expression [32,37]. In fact, basal-like cancer show complex changes in the genomic DNA methylation pattern [22-25]. Here, we report that at least two genomic regions located in the promoters of IL-6 and *CD133 *(*IL-6dist*, *CD133p2 *see Additional file 3 Figure S1 E and F) genes gain methylation in response to IL-6. In both regions, binding sites for putative repressors (IRF-1/2 and ERα) are likely to be present. We therefore we speculate that *IL-6 *and *CD133 *gene transcription can be enhanced by a combination of loss and gain of methylation at genomic regions with opposite functional roles.

As far as protein expression, IL-6 secretion was found to substantially parallel the changes observed at mRNA level. Further, we observed that, similarly to what it has been recently reported [38], CD44 protein was detectable in a low percentage of MCF-7 cells and that CD44 expressing cells became more frequent after the administration of IL-6 for at least 72 hours (Additional file 8 Figure 6S A). Moreover, long term (96 hours) exposure of MCF-7 cells to IL-6 elicited a substantial increase in CD44 expressing cells and in the generation of CD44-expressing mammospheres (Additional file 8 Figure 6S B). Of interest here is the finding that, despite the strict regulation of CD133 mRNA by promoter methylation [30,39], CD133 protein expression was not detected in our experimental models. This was not a completely unexpected finding, because recent published observations on colon CSCs show that CD133 mRNA expression is present in both CD133 positive and CD133 negative colon cancer cells, and that CD133 protein undergoes epitope masking during differentiation [40]. It could be therefore hypothesized that CD133 negative cells expressing CD133 mRNA represent a "primed" population, ready to translate the protein under appropriate environmental conditions or that CD133 mRNA itself exerts regulatory functions. Our preliminary data suggest that CD133 mRNA undergoes cytoplasmic stabilization to be fully expressed in breast cancer cells (D'Uva et al., ms in preparation).

Overall, our data suggest that a remodelling of gene expression toward a basal/stem cell like phenotype may entail a complex reshaping of promoter methylation profile, where a loss of and gain of methylation at different promoter regions occurs (Additional file 9 Figure S7). The above changes are facilitated by the presence of functional p53 impairment. Interestingly, loss of p53 function was recently associated to the shift of cell division from an asymmetric to a symmetric pattern in breast cancer stem cells [41]. Such a phenomenon was proposed as a main mechanism fuelling tumor growth [41]. It could be therefore interesting to investigate whether genes shaping cell division patterns are part of the epigenetic modifications occurring in basal-like tumors [22-25].

This study contributes to recent literature supporting the notion that epigenetic modifications driven by IL-6 are of relevance to determine the gene expression profile of cancer cells [42,43], and we can conclude that IL-6 blockage holds promises as a potential therapeutic strategy to combat breast cancer.

## Abbreviations

NSF: non specific fragment; PD: Primer dimers.

## Competing interests

The authors declare that they have no competing interests.

## Authors' contributions

LDA carried out experimental assays on methylation, manuscript drafting and experimental design. PS carried out RT-PCR analysis and experimental design. GS carried out data on mammospheres, RT-PCR analysis and data analysis. VM participated in cell cultures, viral infections and plasmid amplification. GDU participated in RT-PCR analysis, viral infections and Luciferase assay. PC helped to manuscript drafting and data analysis. MB participated in manuscript drafting, experimental conception and data interpretation. All the Authors read and approved the final version of the manuscript.

## Supplementary Material

Additional file 1 **Table 1**. Primers sequence and conditions for RT-PCR. Click here for file

Additional file 2 **Table 2**. Primers sequence and conditions for MSP-PCR. Click here for file

Additional file 3 **Figure S1 Schematic representation of promoter regions investigated in this study**. a) *IL-6prox *Promoter region; b) *CD133p1 *promoter region; c) *CD44p *promoter region; d) *ERαp *promoter region; e) *IL-6dist *promoter region; f) *CD133p2 *promoter region. Click here for file

Additional file 4 **Figure S2 Reduction of p53 responsive genes mRNA level, increase of IL-6 expression and loss of *IL-6prox *methylation in p53 siRNA-transfected MCF-7 cells**. a) RT-PCR analysis of Bnip3 and p21Waf1 mRNA level in MCF-7 cells transiently transfected with control (scr) or p53-specific siRNA (sip53, 1 μg, 48 h); b) Western blot analysis of p53 protein level and RT-PCR analysis of IL-6 mRNA level in scr/sip53 transfected MCF-7 cells, c) quantitative evaluation of *IL-6prox *MS-PCR analysis in scr/sip53 transfected MCF-7 cells; β-Actin was assessed as quantitative control in RT-PCR and western blot analysis. Click here for file

Additional file 5 **Figure S3 Stat-3 down-regulation by IL-6 blocking antibody**. Western blot analysis of total and phophorylated (P) Stat3 protein level in MCF-7 cells in presence of IL-6 (10 ng/ml 48 h) and p53 D and MDA-MB231 cells exposed to αIL-6 (1.5 μg/ml, 48 h); β-Actin was assessed as quantitative control for Western Blot analysis. Click here for file

Additional file 6 **Figure S4 *CD133p1 *methylation reduces promoter activity, it is reduced in CD133 mRNA expressing MCF-7 S and MDA-MB231 cells, and it is increased by αIL-6 or siIL-6 administration to MDA-MB231 cells**. a) Luciferase assay of CD133p1 reporter (CD133p1-Luc) in presence/absence of IL-6 (10 ng/ml, 24 h) or in presence/absence of SssI methylase (Methylated/Unmethylated vector); RT-PCR analysis of CD133 mRNA level and quantitative evaluation of *CD133p1 *MS-PCR analysis in: b) MCF-7 S and c) MDA-MB231 cells; d) quantitative evaluation of *CD133p1 *MS-PCR analysis in MDA-MB231 cells in presence/absence of αIL-6 (1.5 μg/ml, 48 h) or transfected with scr/siIL-6 (1 μg, 48 h); β-Actin was assessed as quantitative control for RT-PCR analysis. Note that β-Actin of panels b) and c) are reported in Figure 2c and 1a, respectively. Student t test, *p < 0.05; ^#^p < 0.005. NSF: Non Specific Fragment. Click here for file

Additional file 7 **Figure S5 *CD44p *methylation is reduced in CD44 mRNA expressing MCF-7 S and MDA-MB231 cells, and it is increased by αIL-6 or siIL-6 administration to MDA-MB231 cells**. RT-PCR analysis of CD44 mRNA level and quantitative evaluation of *CD44p *MS-PCR analysis in: a) MCF-7S; b) MDA-MB231 cells; quantitative evaluation of *CD44p *MS-PCR analysis in MDA-MB231 cells in presence/absence of αIL-6 (1.5 μg/ml, 48 h) or transfected with scr/siIL-6 (1 μg, 48 h); β-Actin was assessed as quantitative control for RT-PCR analysis. Note that β-Actin of panels a) and b) are reported in Figure 2c and 1a, respectively. Click here for file

Additional file 8 **Figure S6 Long term exposure of MCF-7 cells to IL-6 induces CD44 protein expression and generation of CD44 expressing MCF-7 S**. a) Immunofluorescence analysis of CD44 expression (nuclei are counterstained with DAPI) in adherent MCF-7 cells exposed to 10 ng/ml IL-6 for 72 or 96 h; b) Immunofluorescence analysis of CD44 expression in MCF-7 S generated from cells cultured in presence/absence of IL-6 10 ng/ml for 96 h (scale bars: 75 μm). Click here for file

Additional file 9 **Figure S7 Schematic representation of the data**. IL-6 induces a loss of methylation at *IL-6prox, CD133p1 and CD44p *regions, concomitant with a gain of promoter methylation at *IL-6dist, CD133p2 *and *ERαp *(see Additional file 3 Figure 1S for details on the genomic regions involved). Such changes in promoter methylation pattern associate with an increase in IL-6, CD133 and CD44 expression, and with ERα down-regulation. p53 loss of function, by triggering autocrine IL-6 loop, induces the methylation pattern above, that can also be elicited by exposure to exogenous IL-6. Click here for file
