# Supplementary material for: Epigenetic control of the basal-like gene expression profile via Interleukin-6 in breast cancer cells
Source: Mol Cancer. 2010 Nov 23;9:300. doi: 10.1186/1476-4598-9-300 (PMC3002335; doi:10.1186/1476-4598-9-300)
Supplement: Additional file 1 — Table 1. Primers sequence and conditions for RT-PCR. [file 1476-4598-9-300-S1.PDF]

Your complimentary use period has ended. Thank you for using PDF Complete.

Click Here to upgrade to Unlimited Pages and Expanded Features

Name	Forward 5ô→3ô	Reverse 5ô→3ô	T a ęC	Amplicon (bp)
IL-6	gagaaaggagacatgtaacaagagt	gcgcagaatgagatgagttgt	57	400
CD133	ctggggctgctgtttattattctg	acgccttgtccttggtagtgttg	62	337
CD44	cagcaaccctactgatgatgacg	gccaagagggatgccaagatga	62	323
ERŬ	tgaaagtgggatacgaaaagac	caggatctctagccaggcacat	62.5	410
p53	gccatggaggagccgcagtc	tcagtctgagtcaggccctt	65	1189
Bnip3	cgttccagcctcggtttctattta	cgccttccaatatagatccccaat	56	465
p21Waf	tggggatgtccgtcagaacc	tggagtggtagaaatctctcatgct	59	446
ƃ-Actina	ggcatccacgaaactaccttcaac	agtgatctccttctgcatcctgtc	62	157
