# Supplementary material for: Epigenetic control of the basal-like gene expression profile via Interleukin-6 in breast cancer cells
Source: Mol Cancer. 2010 Nov 23;9:300. doi: 10.1186/1476-4598-9-300 (PMC3002335; doi:10.1186/1476-4598-9-300)
Supplement: Additional file 2 — Table 2. Primers sequence and conditions for MSP-PCR. [file 1476-4598-9-300-S2.PDF]

PDF Complete
Your complimentary use period has ended. Thank you for using PDF Complete.
Click Here to upgrade to Unlimited Pages and Expanded Features

Name	Forward 5ô→3ô	Reverse 5ô→3ô	T a ęC	Amplicon (bp)
IL-6dist U	ggattatagtgtatggttgt	taatcctctaccacaata	52	178
IL-6dist M	ggattatagtgtacggttgc	taatcctctaccgcgata	53	174
IL-6prox U	tttgagtttattgggaat	accaaaaacacctatacca	56	244
IL-6 prox M	ttcgagtttatcgggaac	accgaaaacgcctataccg	56	244
CD133 P1 U	aagggtttaatgtggttgggatgag	acccaccaacacctaaacaacatc	61	169
CD133 P1 M	ggtttaatgcggtcgggatgag	ccctacactcccttcttacaacg	61	114
CD133 P2 U	gggatagaggaagttgtaatggg	aaacccacctcccactacc	52.6	126
CD133 P2 M	gggatagaggaagtcgtaacgg	aaacccgcctcccactacc	58	126
CD44 U	gtttggtttgttattttgtttt	aatcaatctacaccaaactcaac	61	171
CD44 M	cggttcgttattttcgtttc	aatcgatctacgccaaactc	62	168
ERŬ U	ggatatggtttgtattttgtttgt	acaaacaattcaaaaactccaact	53	123
ERŬ M	gatacggtttgtattttgttcgc	cgaacgattcaaaaactccaact	49	121
