# Supplementary figures and images for: Epigenetic control of the basal-like gene expression profile via Interleukin-6 in breast cancer cells
Source: Mol Cancer. 2010 Nov 23;9:300. doi: 10.1186/1476-4598-9-300 (PMC3002335; doi:10.1186/1476-4598-9-300)

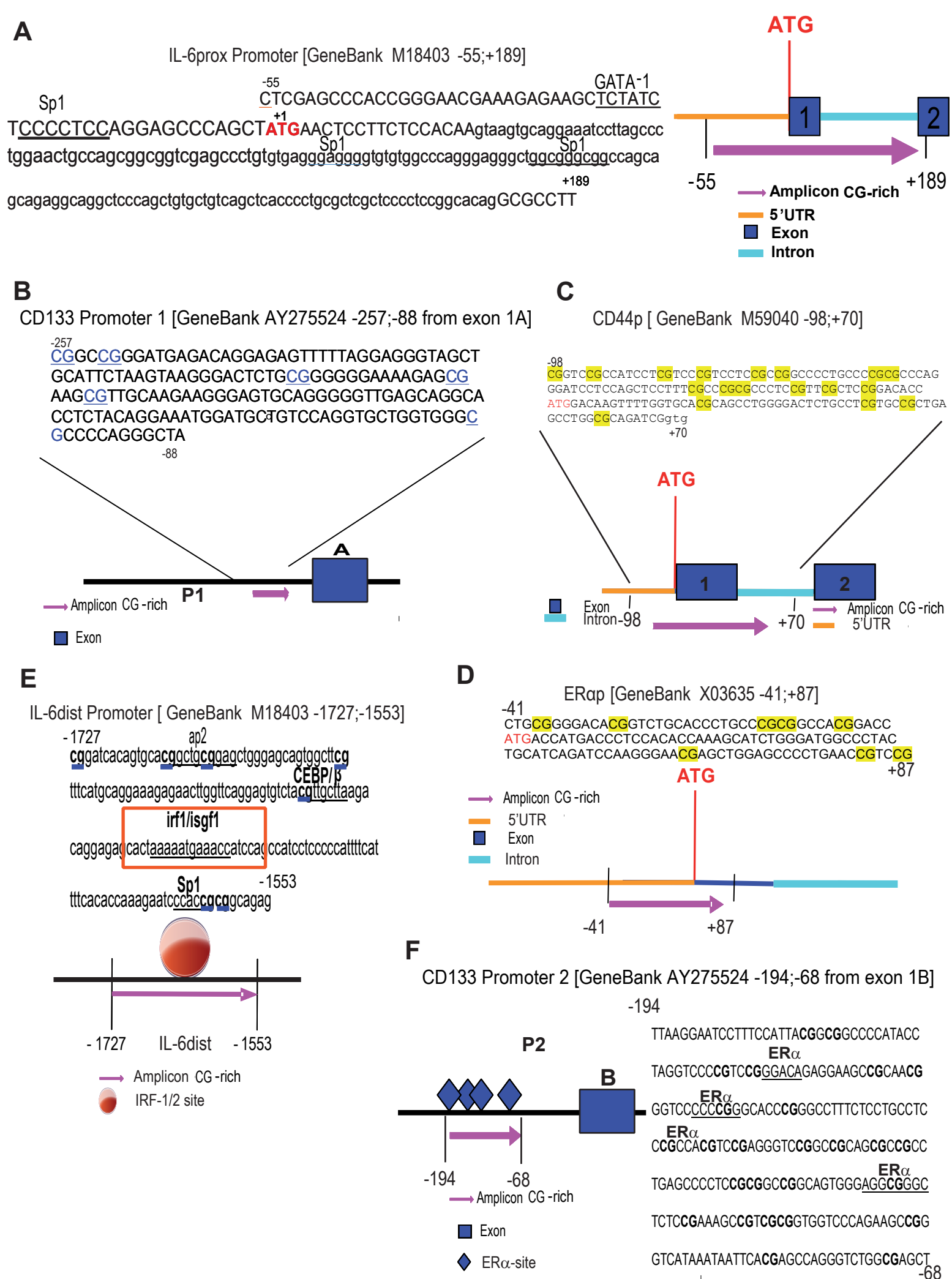

Supplement: Additional file 3 — Figure S1 Schematic representation of promoter regions investigated in this study. a) IL-6prox Promoter region; b) CD133p1 promoter region; c) CD44p promoter region; d) ERαp promoter region; e) IL-6dist promoter region; f) CD133p2 promoter region. [file 1476-4598-9-300-S3.PDF]

## MCF-7

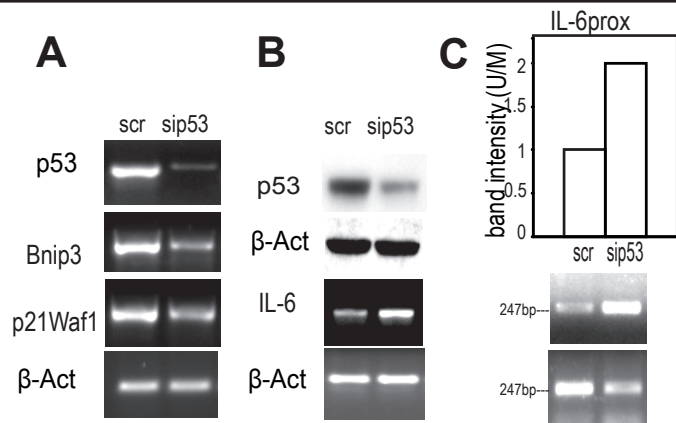

D'Anello et al. Figure S2

Supplement: Additional file 4 — Figure S2 Reduction of p53 responsive genes mRNA level, increase of IL-6 expression and loss of IL-6prox methylation in p53 siRNA-transfected MCF-7 cells. a) RT-PCR analysis of Bnip3 and p21Waf1 mRNA level in MCF-7 cells transiently transfected with control (scr) or p53-specific siRNA (sip53, 1 μg, 48 h); b) Western blot analysis of p53 protein level and RT-PCR analysis of IL-6 mRNA level in scr/sip53 transfected MCF-7 cells, c) quantitative evaluation of IL-6prox MS-PCR analysis in scr/sip53 transfected MCF-7 cells; β-Actin was assessed as quantitative control in RT-PCR and western blot analysis. [file 1476-4598-9-300-S4.PDF]

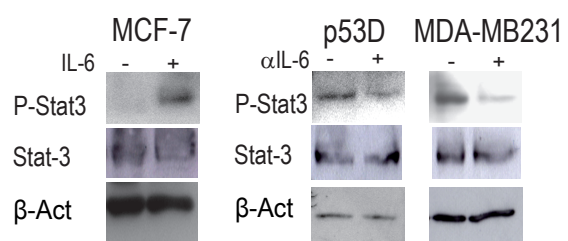

D'Anello et al. Figure S3

Supplement: Additional file 5 — Figure S3 Stat-3 down-regulation by IL-6 blocking antibody. Western blot analysis of total and phophorylated (P) Stat3 protein level in MCF-7 cells in presence of IL-6 (10 ng/ml 48 h) and p53 D and MDA-MB231 cells exposed to αIL-6 (1.5 μg/ml, 48 h); β-Actin was assessed as quantitative control for Western Blot analysis. [file 1476-4598-9-300-S5.PDF]

**A**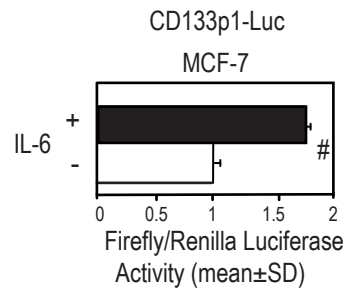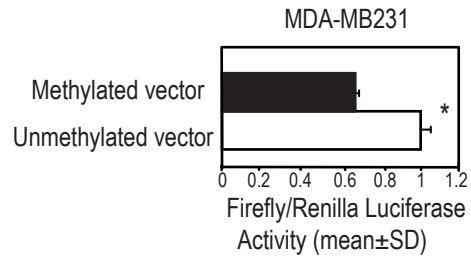**B**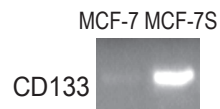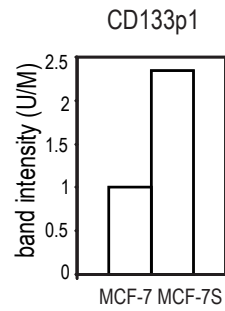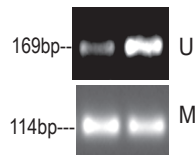**C**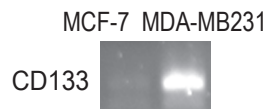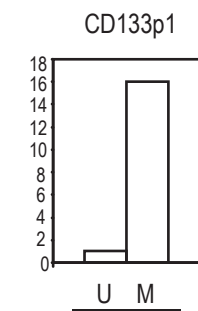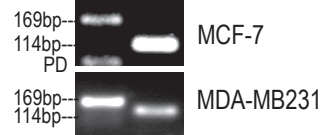**D**

MDA-MB231

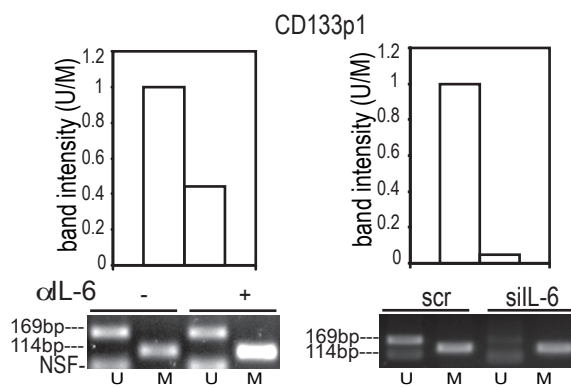

Supplement: Additional file 6 — Figure S4 CD133p1 methylation reduces promoter activity, it is reduced in CD133 mRNA expressing MCF-7 S and MDA-MB231 cells, and it is increased by αIL-6 or siIL-6 administration to MDA-MB231 cells. a) Luciferase assay of CD133p1 reporter (CD133p1-Luc) in presence/absence of IL-6 (10 ng/ml, 24 h) or in presence/absence of SssI methylase (Methylated/Unmethylated vector); RT-PCR analysis of CD133 mRNA level and quantitative evaluation of CD133p1 MS-PCR analysis in: b) MCF-7 S and c) MDA-MB231 cells; d) quantitative evaluation of CD133p1 MS-PCR analysis in MDA-MB231 cells in presence/absence of αIL-6 (1.5 μg/ml, 48 h) or transfected with scr/siIL-6 (1 μg, 48 h); β-Actin was assessed as quantitative control for RT-PCR analysis. Note that β-Actin of panels b) and c) are reported in Figure 2c and 1a, respectively. Student t test, *p < 0.05; #p < 0.005. NSF: Non Specific Fragment. [file 1476-4598-9-300-S6.PDF]

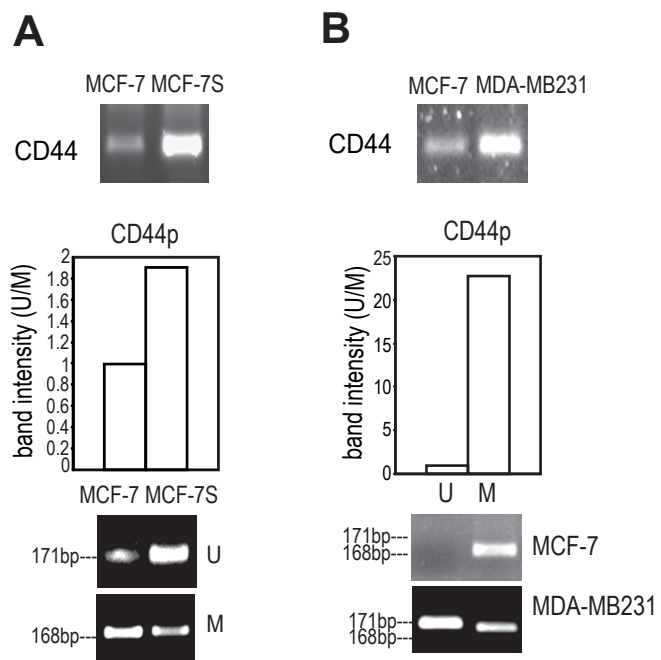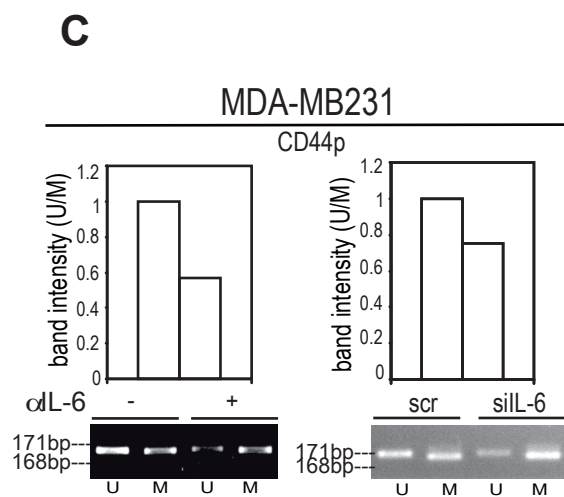

D'Anello et al. Figure S5

Supplement: Additional file 7 — Figure S5 CD44p methylation is reduced in CD44 mRNA expressing MCF-7 S and MDA-MB231 cells, and it is increased by αIL-6 or siIL-6 administration to MDA-MB231 cells. RT-PCR analysis of CD44 mRNA level and quantitative evaluation of CD44p MS-PCR analysis in: a) MCF-7S; b) MDA-MB231 cells; quantitative evaluation of CD44p MS-PCR analysis in MDA-MB231 cells in presence/absence of αIL-6 (1.5 μg/ml, 48 h) or transfected with scr/siIL-6 (1 μg, 48 h); β-Actin was assessed as quantitative control for RT-PCR analysis. Note that β-Actin of panels a) and b) are reported in Figure 2c and 1a, respectively. [file 1476-4598-9-300-S7.PDF]

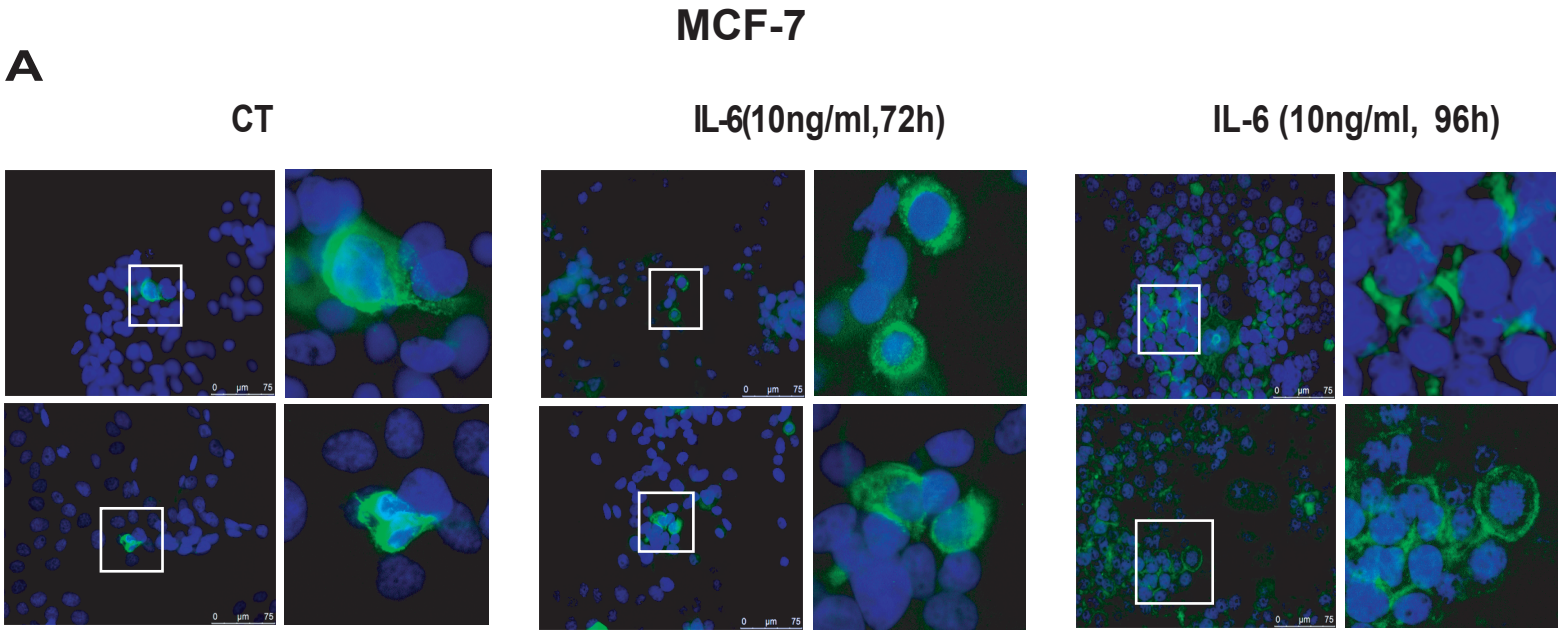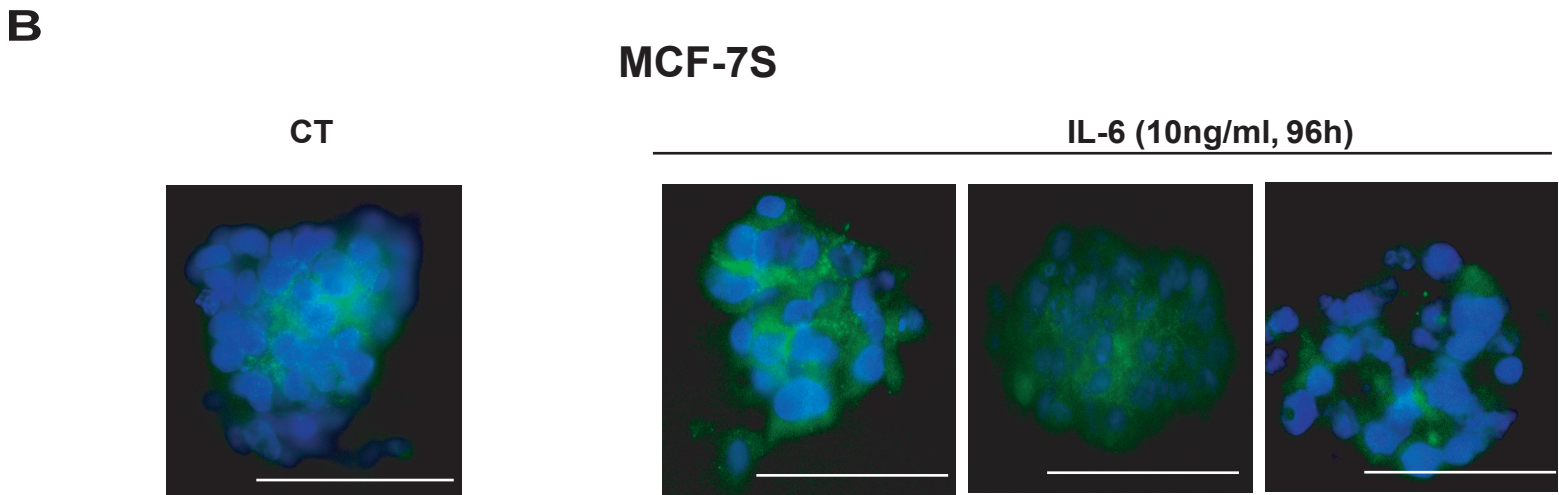

Supplement: Additional file 8 — Figure S6 Long term exposure of MCF-7 cells to IL-6 induces CD44 protein expression and generation of CD44 expressing MCF-7 S. a) Immunofluorescence analysis of CD44 expression (nuclei are counterstained with DAPI) in adherent MCF-7 cells exposed to 10 ng/ml IL-6 for 72 or 96 h; b) Immunofluorescence analysis of CD44 expression in MCF-7 S generated from cells cultured in presence/absence of IL-6 10 ng/ml for 96 h (scale bars: 75 μm). [file 1476-4598-9-300-S8.PDF]
