# Supplementary material for: Epigenetic control of the basal-like gene expression profile via Interleukin-6 in breast cancer cells
Source: Mol Cancer. 2010 Nov 23;9:300. doi: 10.1186/1476-4598-9-300 (PMC3002335; doi:10.1186/1476-4598-9-300)
Supplement: Additional file 9 — Figure S7 Schematic representation of the data. IL-6 induces a loss of methylation at IL-6prox, CD133p1 and CD44p regions, concomitant with a gain of promoter methylation at IL-6dist, CD133p2 and ERαp (see Additional file 3 Figure 1S for details on the genomic regions involved). Such changes in promoter methylation pattern associate with an increase in IL-6, CD133 and CD44 expression, and with ERα down-regulation. p53 loss of function, by triggering autocrine IL-6 loop, induces the methylation pattern above, that can also be elicited by exposure to exogenous IL-6. [file 1476-4598-9-300-S9.PDF]

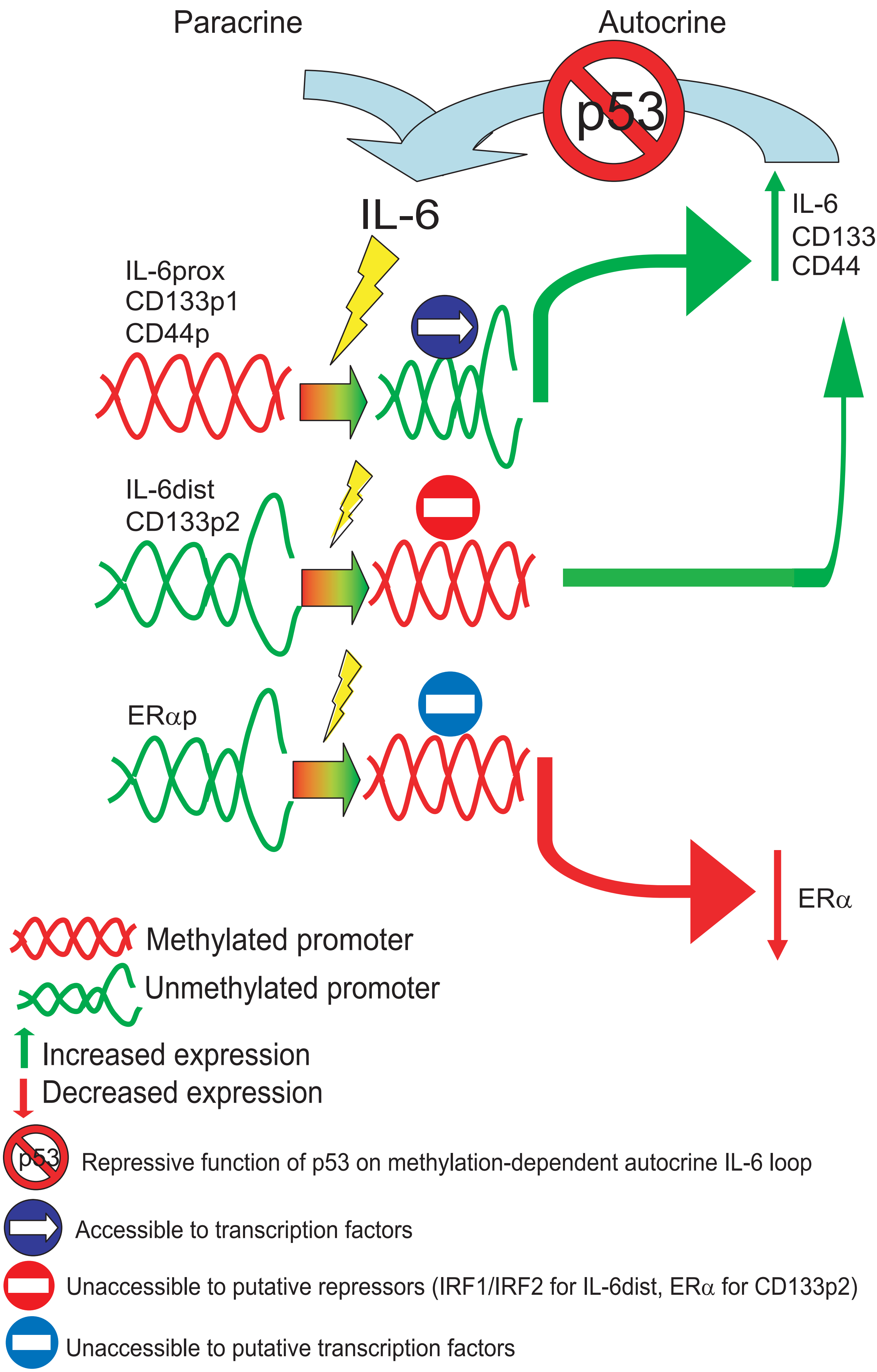

D'Anello et al. Figure S7
